# A Non-coercive Prevention Cascade Using a Cash-Plus Model for Legally Involved Youth: a Multi-method Study of Feasibility, Acceptability, and Engagement Outcomes

**DOI:** 10.1007/s11121-025-01785-z

**Published:** 2025-02-14

**Authors:** Sarah C. Walker, Asia S. Bishop, Juan Gudino, McKenna Parnes, Taquesha Dean, Alya A. Azman, Kristin Vick, Noah Gubner

**Affiliations:** 1 Department of Psychiatry and Behavioral Sciences, University of Washington, 4333 Brooklyn Ave NE, Seattle, WA 98105 USA; 2 School of Social Work and Criminal Justice, University of Washington, Tacoma, USA

**Keywords:** Youth, Criminal legal, Social determinants, Cascade model, Cash-plus

## Abstract

Youth involved in the criminal legal system (YILS) are more likely to experience significant disruptions in social determinants of health. This contributes to, and is reciprocally affected by, ongoing contact with the legal system. Using multiple methods, the current study examined the feasibility and impact of a prevention cascade model designed to universally identify and address early signals of social determinants of health needs including housing and family cohesion using a cash-plus, navigator model. The analysis included 147 youth referred to the Housing Stability for Youth in Courts (HSYNC) program from four juvenile courts in one northwestern state from 2019 through 2022. Analyses revealed youth and families receiving cash-plus were five times more likely to engage in recommended services. Engagement in services across all types of need severity (prevention and crisis) exceeded published benchmarks for other prevention and intervention engagement models. These findings suggest non-coercive services using cash-plus is a highly promising model for connecting YILS and their families to services designed to strengthen social determinants of health.

## Background

Youth involved in the US criminal legal system (YILS) experience significantly worse social determinants of health (SDOH). Nearly 40% of youth involved in the legal system (YILS) will experience housing instability (Chapple et al., 2004; Yoder et al., 2014), higher levels of neighborhood disorganization and violence (Butcher et al., 2015), and higher levels of family conflict (Mowen & Boman, 2018). As homelessness and the failure to access other beneficial SDOHs exert a devastating effect on the physical, behavioral, and psychosocial health of youth (Bender et al., 2014; Hodgson et al., 2013; Medlow et al., 2014), addressing malleable determinants at the point of criminal legal contact provides an opportunity for stabilizing long-term health outcomes (Nordness et al., 2002; Walker & Herting, 2020). The prevention science and medical field is increasingly taking account of social determinants of health in developing system and clinical interventions.

YILS are much more likely than their peers to live in environments degraded by poor social determinants of health, and experience challenges such as homelessness as a result (Young et al., 2014). Social services and prevention and healthcare workers have a limited but meaningful ability to intervene in ways that recognize and address social determinants of health with individual clients. A growing number of studies from the healthcare field demonstrate the risks of failing to consider these social histories and needs when treating specific health outcomes (Iezzoni et al., 2015).

### Homelessness Prevention Among Youth Involved in the Legal System

Social determinants play a key role in the health trajectories of YILS and their risk for continued legal system involvement (Snyder et al., 2016); Bonham et al., 2023). However, few studies with YILS populations have explored the feasibility of integrating a social determinants of health framework into more traditional case management approaches. There is a notable gap in research on the prevention of youth homelessness among YILS among an otherwise rapidly expanding portfolio of federal research on the prevention of youth homelessness among foster care youth (Cole et al., 2021), within school settings (Edgerton, 2023), and as a health benefit for medical services. A previous scoping review found that the literatures of YILS intervention and social determinants of healthcare are not well-integrated (Almquist & Walker, 2022).

### Prevention Services Cascade in Youth Legal Services

The cascade of care framework could be a promising model for adopting a SDOH-integrated approach for YILS (Williams et al., 2019). This framework describes stages of care, beginning with universal screening of a focus population and service linkages through the continuum of intervention. Previous applications of the cascade of care within the juvenile legal system focus primarily on substance use identification and service referral, with phases including universal screening of probation-involved youth, referral to treatment, linkage to treatment by means of incentives, reminders or navigators, and completion of treatment (Aalsma et al., 2021; Scott et al., 2019). These approaches show modest support for the application of a cascade framework in juvenile justice settings (Elkington et al., 2020). However, underlying economic and family stressors (Elkington et al., 2020) and innovation burnout among court and probation staff (Goroll, 2020) suggest future models should focus on strengthening cross-sector coordination, addressing underlying family needs, and streamlining innovations to improve uptake.

### Service Navigation, Cash Transfers

Family cohesion interventions, economic assistance, and short-term housing can improve youth housing stability and SDOH (Almquist & Walker, 2022). Strengthening the cascade framework to increase engagement in these services is expected to improve individual- and population-level SDOH outcomes. Specifically, adding service navigation (supporting a client to assess and engage with community resources) with cash support could strengthen cross-sector coordination and service engagement outcomes. When navigation is delivered by individuals with lived expertise, for example by community health workers or peers, this model can also provide direct therapeutic benefit by providing hope and emotional support to clients (Fuhr et al., 2014). Connecting cash transfers to social service program, or “cash-plus,” further increases the likelihood of obtaining service engagement and positive health benefits (Roelen et al., 2017; Veras Soares et al., 2016).

The current study examined the feasibility of a prevention cascade model for YILS designed to address social determinants of health with a focus on housing stability and family cohesion. The feasibility study evaluated the observed cascade and compared the successful retention of clients at each point using benchmarks from other service engagement interventions with YILS populations. The study then evaluated client demographic factors and the use of cash-equivalent support on successful service engagement within the cascade in a cash-plus model.

## Methods

### Housing Stability for Youth in Courts (HSYNC) Model

HSYNC was designed to focus on addressing malleable social determinants of health with the aim of preventing the onset or ongoing episodes of housing instability and strengthening family cohesion in four Washington state counties (Walker et al., 2018). The HSYNC cascade model proceeded from universal screening to referral to the navigator, brief meeting, triage, service referral, cash-equivalent support, and service engagement.

#### Universal Screening

The intended populations for screening were YILS assigned to probation supervision. The unit of intervention was the family, although the program served youth or caregivers individually if either one was not willing or available to participate. To minimize burden on court staff, the HSYNC approach used two items from an assessment already in use, the Positive Achievement Change Tool, as universal screening indicators: (1) any prior history of runaway and (2) high levels of family conflict in the home (Mowen & Boman, 2018).

#### Referral to Navigator

Following identification, court staff approached the youth or caregiver (“client”) about the HSYNC program. If the client agreed to a referral, staff completed a navigator referral form. Forms were fillable Word documents and were emailed through secure servers to the navigator.

#### Brief Meeting

Following referral, a service navigator reached out to the client (youth or caregiver as recommended by the referral) to introduce themselves and describe program services, emphasizing that engaging in the HSYNC program was unrelated to any legal requirement. Navigators were individuals hired for the project who had lived experience being involved with or supporting close friends or family who were involved with the criminal legal system, as well as professional experience in case management. Brief meetings typically occurred over the phone but occasionally occurred in-person at the court.

#### In-Depth Meeting and Triage

Navigators were trained to use a conversational, client-centered approach to gather information about client needs. The assessment of needs was informed by a 15-item written triage tool developed for HSYNC and adapted from a bespoke housing assessment and the Family Adaptability and Cohesion Evaluation Scale II family cohesion scale (Olson et al., 1983). Total scores were calculated by summing across domains, resulting in one of five categories of recommended prevention intensity: none, low (selective prevention), moderate (selective to indicated prevention), high (tertiary prevention), active housing need. Results of the assessment were used to recommend family support and SDOH resources using a stepped care framework for prevention services.

#### Service Referral

Navigators drew from a compiled list of services in the region to make tailored recommendations to the client based on the client’s prevention intensity score and client preferences (Gilbert et al., 2023). Low-intensity prevention services included primarily social service referrals (e.g., utility support), self-guided resources, including online parenting courses, or mediation to resolve caregiver-youth conflict provided by the navigator. Moderate-intensity prevention services included programs typically designated as secondary prevention programs. These included parenting-focused groups such as Guiding Good Choices (Catalano & Hawkins, 1996) and Strengthening Families (Kumpfer et al., 2015). High-intensity prevention services included in-home services designed to prevent out-of-home placement or running away. These typically included referrals to the state wraparound program for complex mental health needs, Functional Family Therapy (Alexander et al., 2013), or family systems therapy.

#### Housing Services

Youth who were experiencing homelessness at the time of referral or were assessed to have an imminent risk for homelessness were referred to housing coordination services. These services were provided in house for two of the HSYNC programs, and operated externally for the other two programs.

#### Cash and Equivalent Supports

In addition to service referral, participation in HSYNC included the opportunity to receive cash-equivalent support with instrumental, short-term needs. Navigators were encouraged to spend a minimum of $50 on moderate and high prevention cases to support engagement in the HSYNC program and referred services. No restrictions were placed on navigators’ discretion in offering funds to client at any prevention level and no restrictions were placed on an upper limit of allowable funds that could be spent on one client. Cash-equivalent support was generally provided once and was offered on top of offers to pay for coffee and lunches as part of HSYNC client meetings. Some of the cash-equivalent support clients received included essentials such as food, toiletries, groceries, clothing, transportation, and phone bill payments.

#### Service Engagement

Navigators reached out to clients biweekly or more often if desired by the client to support successful service engagement. The period of engagement lasted up to 3 months and cases could be closed earlier if clients were successfully engaging in a recommended service and did not indicate a need for more support, or if clients were unresponsive after five outreach attempts.

#### Navigator Coaching and Fidelity Monitoring

Navigators participated in monthly group coaching and monthly individual check-ins with a trained doctoral research assistant. Monthly individual check-ins were guided by a semi-structured checklist. The graduate assistant received these sessions at biweekly project meetings and a PhD-level researcher reviewed check-in notes periodically to assess quality and provide feedback on approach and documentation.

### Data Sources

Data for quantitative analyses for observational analysis and regression models were obtained from navigator case tracking files developed by the research team and completed by the program navigators under the research teams’ supervision. To ensure data integrity, the research team conducted periodic data audits on case files. This involved obtaining de-identified data from the navigators on a subset of cases. De-identified case files were transferred to the research team at the end of the study period.

Data for qualitative analyses related to client satisfaction were obtained from structured interviews conducted with caregivers and youth involved in the HSYNC program. HSYNC program participants were informed about the opportunity to participate in the interviews through flyers provided to the navigators. Qualitative analysis for coding engagement was obtained from written navigator case notes. All research activities were approved by the University of Washington Institutional Review Board.

### Analytic Strategy

To assess the feasibility of the cascade, the percent of clients retained at each level was calculated. Chi-square tests were also calculated to assess between and within group differences for service referral and engagement type. Multivariate regressions were conducted to assess the relationship between independent variables (demographic and cash-equivalent support) and service engagement outcomes, controlling for baseline youth characteristics.

### Qualitative Analysis

Youth and caregiver interviews were analyzed following a thematic analysis approach (Braun & Clark, 2006). This included an inductive, iterative process of (1) developing a coding framework and coding the data; (2) using research team discussions to enhance credibility and trustworthiness (i.e., researcher triangulation; Miles et al., 2020); and (3) condensing codes and naming themes. Two members of the research team manually coded the data using the established coding framework. Disagreements were discussed and negotiated among the coders until consensus was reached. Theme titles were developed to describe the focus of each set of codes.

### Independent Measures

Client demographics included client race/ethnicity, gender and sexual orientation, and age. The case tracking files captured information on the type of cash-equivalent support (e.g., gift card, food). Given the small sample size, this was transformed to a binary 0/1 indicating whether any support was provided. Whether the client was referred to services was captured with a binary variable (no/yes) and a categorical variable indicating (1) family-based services; (2) housing services; (3) substance use treatment services; or (4) other services.

### Outcomes

Engagement (low, moderate, high) was derived from qualitatively coding case note files. A coding rubric was developed by a PhD-level researcher after reviewing five cases. Two study research assistants were trained on the coding rubric. Low engagement was defined as zero to one response to navigator outreach. Moderate engagement was defined as between three and five responses to navigator outreach. High engagement was defined as over five responses to navigator outreach. Responses were defined as interactions (phone calls, meetings).

Engagement in services was derived from navigator entry in binary case management data variable (yes/no) and was cross-checked by the research team with case notes to confirm accuracy.

## Results

Results are presented first for the cascade findings, examining the total referred, sample characteristics, and retention across service points. Next, the results examine characteristics of clients and services associated with successful service engagement.

### Participants

A total of 147 youth were referred to HSYNC from 2019 to 2022. All 147 youth referred to caregivers opted into the qualitative interviews. Within the 147 youth, the mean age was 16.34 years. Roughly a third of the sample identified as White (37%), followed by Black (14%), Multiracial (8%), and American Indian/Native American (5%). Small numbers of youth identified as Asian (*n* = 1), Native Hawaiian/Pacific Islander (*n* = 1), or “other” (*n* = 4). Twenty youth (*n* = 20) identified as Hispanic across racial categories (14%). Participants were slightly more likely to be male (40%), followed by female (32%), with small numbers of youth identifying as non-binary (*n* = 1), trans man (*n* = 2), and trans woman (*n* = 1) and the remaining youth (25%) declined to answer. Youth were most likely to identify as heterosexual (40%), with 8% reporting as LGBTQ + and the remaining sample declining to answer. We are unable to report demographic characteristics for the interview sample, as this information was destroyed prior to analysis in accordance with the study’s IRB-approved data retention policy. The measure of family cohesion using the assessment yielded average scores suggesting most youth had only moderately problematic family relationships between youth and their parents/guardians (sample mean = 5.03) and no meaningful differences in family cohesion among youth with identified prevention vs. crisis housing needs (prevention youth mean = 5.09, crisis youth mean = 4.97).

### Coverage in the Prevention Cascade

**Fig. 1:**
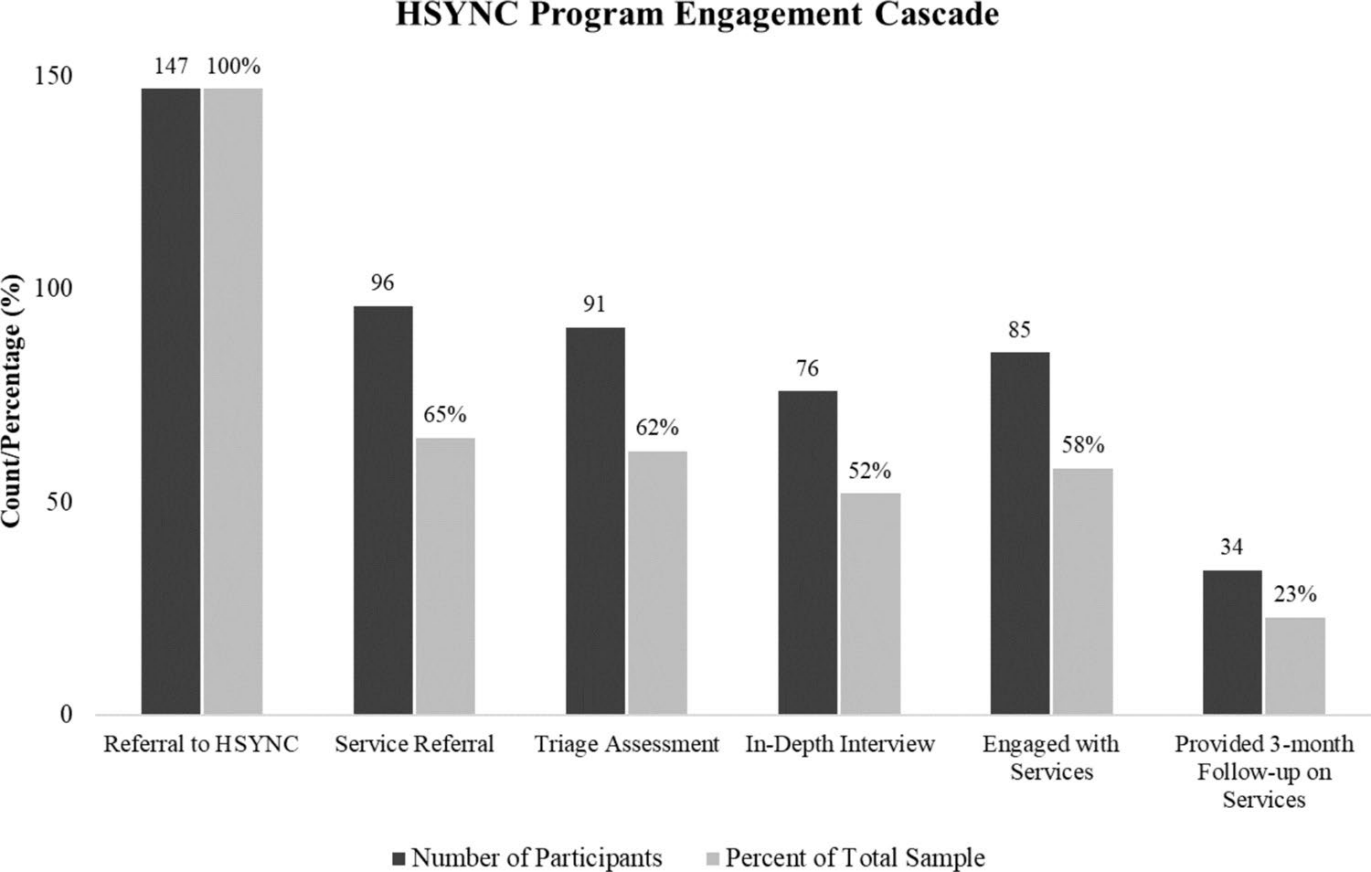
HSYNC program engagement cascade

As demonstrated in Fig. 1, we observed a loss of 51 participants from navigator referral to service referral (35% loss). Among those who received a service referral, most received a triage assessment and score (91/96, 95%), and fewer received an in-depth meeting with the navigator (76/96, 79%). We found that the steps in the observed cascade diverged slightly from the steps in the expected cascade. As described in the methods, the expected cascade proceeded from court referral to initial navigator contact, in-depth meeting, triage score, referral to services, and monitoring service engagement. In the observed cascade, a slightly higher number of participants engaged with services (58%) than completed an in-depth meeting with the navigator (52%), suggesting that the in-depth interview was not always necessary for successful service referrals following initial navigator contact. Out of the total sample referred to the program (*N* = 147), a little over half of clients (58%, *N* = 85) engaged with recommended services. As this slightly underperforms compared to the rate reported for probation monitored service referral (68%; Wasserman et al., 2021), the findings suggest the navigator program was roughly as effective as legally mandated services in achieving initial service engagement. However, only 23% of participants could be re-contacted by navigators 3 months after referral, leaving the status of more sustained engagement unknown.

### Measures of Navigator and Service Engagement

Level of engagement with the navigator was calculated for those participants with case note data (*N* = 114). The majority of participants were moderately (*N* = 48, 42%) or highly (*N* = 39, 34%) engaged with the navigator (Table 1). We observed meaningful differences in the level of navigator engagement between those needing prevention vs. crisis services with 78% of prevention cases vs. 75% of crisis cases having moderate to high levels of engagement. Service engagement, having visited at least one service following navigator referral, was higher among crisis cases (59% vs. 40% prevention cases).

**Table 1:** Client demographics

	Full sample (*n* = 147)	
	Prevention (*n* = 50)	Crisis (*n* = 64)
Family conflict score (mean)	5.09 (2.53)	4.97 (2.81)
Cash support applied (% yes)	5 (10.0)	14 (21.9)
Assessment (% yes)	26 (52.0)	30 (46.9)
Family services referred % (yes)	25 (50.0)	31 (48.4)
Any services referred (% yes)	36 (72.0)	59 (92.2)
Any services accepted (% yes)	22 (44.0)	31 (48.4)
Navigation duration (days, mean)	93.03 (60.85)	98.11 (87.63)
Contact attempts (mean)	8.77 (6.10)	12.05 (13.37)
Successful contacts (mean)	5.54 (4.70)	8.74 (10.59)
Service engagement (% yes)	20 (40.0)	38 (59.4)
Engagement level		
Unengaged (%)	8 (16.0)	14 (21.9)
Low (%)	3 (6.0)	0 (0.0)
Moderate (%)	25 (50.0)	23 (35.9)
High (%)	14 (28.0)	25 (39.1)
3-month follow-up (% yes)	12 (24.0)	22 (34.4)

### Match Between Case Type and Service Referral

A chi-square test of independence was performed to examine the relationship prevention cases vs. crisis cases and overall service referrals and housing service referrals. A significantly higher percentage of crisis cases received any service referral compared to prevention cases, *χ*^2^ (1, *N* = 114) = 8.24, *p* < 0.01. Similarly, a higher percentage of crisis cases received housing service referrals compared to prevention cases, *χ*^2^ (1, *N* = 94) = 13.62,* p* < 0.001 (Table 2).

**Table 2:** Summary of chi-square results

Variables	*χ*^2^	*df*	*p*-value	*N*
Overall service referrals	8.24	1	**0.004**	114
Housing service referrals	13.62	1	** < 0.001**	94
Service engagement within prevention cases				
Housing service referrals	0.70	1	0.404	35
Family service referrals	3.75	1	0.053	38
Other service referrals	1.05	1	0.305	20
Service engagement within crisis cases				
Housing service referrals	4.45	1	**0.035**	59
Family service referrals	5.47	1	**0.019**	49
Other service referrals	1.24	1	0.265	30

Values in bold indicate statistical significance exceeding *p* < .05

Associations between service referrals and overall service engagement were examined within prevention cases and crisis cases (Table 3). Within prevention cases, there were no differences in service engagement based on referral to housing services, referral to family services, or referral to other services. However, a significantly higher percentage of individuals referred to housing services reported overall service engagement compared to individuals not referred to housing services, *χ*^2^ (1, *N* = 59) = 4.45, *p* < 0.05. Similarly, a higher percentage of individuals referred to family services reported overall service engagement compared to individuals not referred to family services, *χ*^2^ (1, *N* = 49) = 5.47, *p* < 0.05. Differences in service engagement based on other service referrals were not observed.

**Table 3:** Multivariate regression associations with service engagement

	Unstandardized *B*	Odds ratio	Standard error	*p*-value	95% CI
Family services accepted (yes)					
Gender (female/non-binary/trans)	− 0.52	0.59	0.47	ns	[− 1.30, 0.25]
BIPOC	0.57	1.76	0.53	ns	[− 0.30, 1.44]
Caregiver as primary contact	− 0.35	0.70	0.52	ns	[− 1.22, 0.51]
Triage	0.34	1.41	1.07	ns	[− 0.19, 0.87]
Cash support	− 0.54	0.58	0.59	ns	[− 1.51, 0.43]
Service engagement (yes)					
Gender (female/non-binary/trans)	0.12	1.12	0.33	ns	[− 0.43, 0.66]
BIPOC	1.15	**3.14**	0.44	0.009	[0.43, 1.86]
Caregiver as primary contact	0.52	1.68	0.41	ns	[− 0.16, 1.20]
Triage	0.24	1.27	0.16	ns	[− 0.03, 0.50]
Cash support	1.58	**4.83**	0.61	0.010	[0.58, 2.57]

Values in bold indicate statistical significance exceeding *p* < .05

### Factors Associated with Service Engagement

Clients who engaged in any service, inclusive of housing, family intervention, or social services, were more likely to be non-White (Black, Indigenous Person of Color, *OR* = 3.14, *p* < 0.01, three times more likely than White clients). Clients who received a material or cash support (*OR* = 4.83, *p* < 0.01) were nearly five times more likely than those not receiving support to engage in services. Gender and whether clients received an in-depth meeting with the navigator were not meaningfully or significantly predictive of service engagement. Clients in which the caregiver was the primary client were modestly more likely to engage in services, but this trend was not statistically significant.

### Youth and Caregiver Perceptions

Three themes emerged from interviews with nine youth and six caregivers. Themes highlighted key features of the program that were important for participant well-being and success, as well as commonly identified challenges to program engagement and suggestions for improvement. Table 4 presents findings from the thematic analysis, including exemplar quotes.

**Table 4:** Youth and caregiver perceptions of the HSYNC program

Theme	Codes	Frequency of codes by group	Example quotes
Theme 1: quality of support and resources	Navigator role and relatability	Youth = 9 Caregiver = 5	“She [navigator] has been great […] she’s been offering resources and she communicates with me and so that just makes it easier to understand and of course she’s knowledgeable and I’ve seen her get in touch with someone she knows would be helpful. She does it right on the spot, doesn’t send me something later.” (Caregiver 1015)
	Emotional support	Youth = 5 Caregiver = 3	“She [the navigator] did the right thing by offering me emotional support which I needed at the time.” (Youth 2030)
	Information and resources	Youth = 9 Caregiver = 5	“She sent me a whole resources page via email of like jobs, or agencies that can help you like job connections, and things through the community like different agencies that can maybe help me upgrade my resume, get more skills, get me in the door to a job eventually.” (Caregiver 1019)
Theme 2: challenges with service engagement	Individual: engagement	Youth = 2 Caregiver = 2	“Just from my person and who I am […] I don’t ask for help when I need it. So the help was definitely there and it was helpful, it’s just […] I guess I didn’t allow myself to access the help.” (Youth 2018)
	Contextual: family	Youth = 2 Caregiver = 0	“I want to meet more but I can’t because I’m taking care of my brothers while my parents aren’t home […] So that concern wasn’t taken as much as I wanted […] I wanted to meet and to meet more but I guess, like, it’s not possible because of other people’s schedules and such.” (Youth 2018)
	Contextual: juvenile court	Youth = 0 Caregiver = 1	“I mean sometimes you feel like just because you have a family member or a child who, you know, went through the court system that people look down on you or that you’re a bad parent or that your children are bad. But there was none of the judging, there was none of that.” (Caregiver 1019)
Theme 3: navigator and family involvement are critical for program effectiveness	Navigator involvement	Youth = 4 Caregiver = 2	“I think it would be more often. Getting in contact more often.” (Youth 2034)
	Family involvement	Youth = 3 Caregiver = 1	“I think there needs to be more connection […] being able to make sure parents can connect with their children and children know that they can be heard. And I think introducing parents in that whole connection, I think it means the world.” (Caregiver 1001)

Youth *n* = 9; caregiver *n* = 6

#### Theme 1: Quality of Support and Resources Provided

All youth and most caregivers were satisfied with the support they received and believed the information and resources offered by the navigator were helpful. Specifically, participants reported being treated well by their navigator and felt that their navigator was relatable, understanding, and served a helpful role in their lives. Participants who reported receiving emotional support also commented on their ability to talk to their navigator about prior experiences of adversity and trauma and indicated that their navigator provided emotional support during a time of need. Participants reported a range of information and resources offered either directly from the navigator or via referral to community-based agencies.

#### Theme 2: Service Engagement Challenges

Contextual factors at the family and court levels impacted program engagement. Youth described how family obligations and family conflict made it difficult to successfully engage in the program. One caregiver noted that their experience with the program differed in a positive way from prior experiences they had with the juvenile court.

#### Theme 3: Navigator and Family Involvement Are Critical for Program Effectiveness

Youth and caregivers provided suggestions for program improvement while also noting aspects of the program they thought worked well. Navigator and family involvement were identified as important for program effectiveness by most youth and caregivers. From the youths’ perspective, ensuring regular and/or frequent contact with the navigator and including parents in the process were identified as important for program effectiveness. Caregivers noted two suggestions for program improvement, including increasing navigator involvement in the service linkage process and ensuring services are tailored to the family’s needs.

## Discussion

The HSYNC program was developed to strengthen key social determinants of health, including housing and family cohesion, for legally involved youth and their families. The current study found that the program achieved benchmarks for feasibility and acceptability, and demonstrated the value of a cash-plus approach for supporting successful service engagement for prevention and intervention/crisis services. These findings are notable within the literature of the youth criminal legal system as HSYNC services were offered outside of probation requirements or supervision. The study provides promising evidence for implementing a cascade of care outside of legal surveillance using a client-centered approach.

A key finding of the study was the strong, positive association between cash-plus and service engagement. Those receiving any type of cash assistance were five times more likely to engage in services recommended by the HSYNC navigator. This finding was observed despite the unconditional nature of the cash-incentive; clients were provided support with basic needs regardless of engagement in other recommended services. Our finding is consistent with studies of conditional and unconditional cash transfer (Robertson et al., 2013), in which the value of conditional cash transfer was not clearly superior to unconditional cash transfers in child outcomes of interest to the study, e.g., child school engagement (Robertson et al., 2013) and HIV prevention (Baird et al., 2012). This is promising for scaling cash-plus programs in public social services, as unconditional cash transfer programs are easier to implement and monitor (Robertson et al., 2013). Our study did not capture data suitable for exploring why cash-plus was successful in improving service engagement outcomes. The broader literature hypothesizes that cash-plus programs work by (1) stabilizing short-term needs and enabling participation in skills-development programs and (2) providing motivation for engaging in cash-plus projects (e.g., Rogers et al., 2024). More research is needed to test this hypothesized pathway, generally, and for legally involved youth, specifically.

We also observed a comparable rate of service engagement for the HSYNC participants to those reported in other YILS service engagement studies using a cascade model. For example, in a study of a service cascade for substance use services within juvenile probation, Wasserman et al. (2021) found that more than half of youth were identified as needing treatment from screening, 1/5th were successfully referred to treatment, 68% initiated, and half of those who initiated remained in care. In the present study, 58% of HSYNC clients initiated services following referral. In Wasserman’s study, youth under higher levels of supervision (vs. those receiving diversion) were five times as likely to be successfully referred to treatment from assessment. In contrast, services referral in HSYNC occurred outside of legal supervision, suggesting the referral rates for HSYNC may exceed those for referrals from diversion services without cash-plus components (Belenko et al., 2017).

We observed more successful service engagement among those clients who had housing needs over those classified as needing community family prevention services. This is consistent with other literature documenting the challenges of engaging families into voluntary prevention services (Filion et al., 2020; Putnam-Hornstein et al., 2021; Randolph et al., 2009). At the same time, service engagement rates in HSYNC for those classified as prevention (40%) exceeded rates observed in voluntary, prevention-oriented services for parents across a variety of health areas generally (3–35%; Smokowski et al., 2018), and families at risk of child maltreatment specifically (18%; Putnam-Hornstein et al., 2021). Elements of HSYNC are consistent with predictions of Randolph et al.’s (2009) framework for parent engagement in prevention services, which identified five elements (termed *cues to action*) important to parents’ interest in engaging in prevention. These include susceptibility (perceived problem), value (perceived benefit), benefits and barriers (worth the effort), expectation (services will help), and self-efficacy (barriers are surmountable). Youth and parent interviews suggest the navigator was a valuable intermediary for the family in building these cues by responding directly to whatever the family perceived as a need. This appeared to strengthen motivation to engage in services.

### Limitations

This study is limited in its ability to draw conclusions about causality as a non-experimental, controlled design. As a result, we cannot state with certainty that the observed findings, and particularly the associations between cash support and BIPOC youth with higher levels of service engagement, are not due to other confounding factors. At the same time, the consistent quality oversight and support provided to sites suggests that differences were likely not due to meaningful differences in fidelity. Strengths of the study included the multi-county sample, a natural implementation environment, and use of multiple sources of data.

### Conclusion

The current study found promising effects for using cash-plus as an alternative to legal surveillance to engage youth and their families into community-based services appropriate for addressing housing stability, family cohesion, and other social welfare needs. Cash-plus increased successful service engagement five times over traditional case management approaches to service referral and engagement. Future studies are needed to study the long-term effects of this model on housing stability and recidivism.

## Data Availability

Data is available from the study authors on request.
